# Median Arcuate Ligament Syndrome: Management and Literature Review

**DOI:** 10.7759/cureus.28889

**Published:** 2022-09-07

**Authors:** Okelue E Okobi, Belinda A Afuda, Maureen Boms, Chinwendum U Ekpemiro, Nneka J Umeh, Chukwudike G Nnaji, Nkemputaife P Onyechi, Oluwatobi G Faderin, Jennifer C Chiji-Aguma, Eboigbe Stephen, Clifford O Amadi

**Affiliations:** 1 Family Medicine, Lakeside Medical Center, Belle Glade, USA; 2 Family Medicine, Humber River Hospital, Toronto, CAN; 3 Clinical Research, University of Alabama at Birmingham, Birmingham, USA; 4 Surgery, Federal Medical Centre, Umuahia, NGA; 5 General Medicine, Brooklyn Queens Nursing Home, New York, USA; 6 Internal Medicine/Family Medicine, Windsor University School of Medicine, Chicago, USA; 7 Internal Medicine, University Hospitals Cleveland Medical Center, Cleveland, USA; 8 Family and Community Medicine, Olabisi Onabanjo University Teaching Hospital, Sagamu, NGA; 9 Internal Medicine, All Saints University Dominica – School of Medicine, Roseau, DMA; 10 General Practice, Lagos University Teaching Hospital, Lagos, NGA; 11 Radiology, Nottingham University Hospitals NHS Trust, Nottingham, GBR

**Keywords:** weight loss, recurrent vomiting, postprandial vomiting, nausea, persistent vomiting, recurrent upper abdominal pain, celiac axis compression syndrome, unexplained abdominal pain, median arcuate ligament syndrome, median arcuate ligament release

## Abstract

Pain, nausea, vomiting, weight loss, diarrhea, and fatigue are common symptoms of several upper gastroenterological illnesses. However, the presence of unexplained recurring postprandial abdominal pain and vomiting increases the possibility of median arcuate ligament syndrome (MALS). MALS is an uncommon illness characterized by postprandial vomiting, abdominal pain, and weight loss. The compression of the median arcuate ligament on the celiac trunk and/or its surrounding celiac nerve plexus may explain this disease phenomenon. Comprehensive workup for other etiologies may be unrevealing except for the compression of the celiac trunk identified in imaging studies and, perhaps, occasional arterial flow rates in sonography studies in some severe cases. Due to the overlapping symptoms of upper gastroenterological disorders, misdiagnosis may be widespread. Therefore, it is essential to consider MALS while examining a patient with upper gastrointestinal disease. In this case series, we present two cases of MALS with similar clinical trajectories and differences in diagnostic techniques.

## Introduction

In patients with recurrent upper gastrointestinal postprandial pain, postprandial vomiting, and weight loss, the differential can be broad, including mechanical, inflammatory, infective, carcinogenic, ischemic, or obstructive causes. Median arcuate ligament syndrome (MALS) may present similarly to the other various etiologies of upper abdominal pain. Diagnosis can be challenging and is usually achieved by exclusion, incidental finding, or a high index of suspicion. However, there may be more specific symptoms that may raise concern, such as postprandial pain accompanied by vomiting that does not correspond with clinical findings. The mechanisms to explain these associated symptoms are still emerging [1-3]. Accurate diagnosis is key to reducing time and resources, establishing a cure, and restoring a patient’s quality of life. While a high index of suspicion remains the clinical compass for diagnosis, imaging modalities and sonographic tests remain the gold standard for increasing accuracy in establishing a diagnosis [4-9]. Thorough knowledge of anatomy and the historical perspective of the syndrome will help reinforce clarity about its everyday presentation. Due to the overlapping nature of symptoms of upper gastroenterological diseases, particularly gastritis and peptic ulcer diseases, the risk of misdiagnosis is high [4-9]. Therefore, it is essential to consider MALS when evaluating a patient with these upper gastroenterological symptoms. In this case series, we describe two patients who presented with recurring postprandial nausea, vomiting, epigastric discomfort, and weight loss, as well as their clinical and therapeutic trajectory. The risk factors remain unclear as more knowledge and increasing diagnoses of this disease evolve [4-6]. The disease has been predominantly reported in females [6]. Numerous etiologic theories exist for this illness, with the possibility of a combination, due to the variety of anatomic etiologies. These include a “high point of origin” of the celiac artery compressed by a diaphragmatic crus or a median arcuate ligament (MAL) that is anatomically located normally. It could also have a diaphragmatic or normal origin for the celiac artery with an outstretched MAL. It has also been proposed that the superior mesenteric ganglia compress the celiac trunk and/or that there are large bilaterally fused celiac ganglia [4,7-9]. Surgery has consistently been reported as a potential management option. If not diagnosed and managed appropriately, common complications such as electrolyte imbalance and its manifestation, prodromes of malnutrition, and possible psychosomatic disorders such as anxiety may arise [4-9].

## Case presentation

Summary of cases

Patient characteristics, investigative timelines, and findings are presented in Table 1.

**Table 1:** Patient characteristics.

Characteristic	Patient 1	Patient 2
Age	36	29
Ethnicity	White	White
Gender	Female	Female
Body mass index (in kg/m^2^)	28	32
Predominant clinical presentation	Recurrent postprandial vomiting, nausea, abdominal pain, and weight loss	Recurrent postprandial vomiting, nausea, abdominal pain, and weight loss
Severity of symptoms	Mild	Mild-moderate
Duration of recurrent symptoms	Five months	Approximately three years
Endoscopic findings	Mild gastric mucosal erythema	Negative
Helicobacter pylori tests	Negative	Negative
Amylase and lipase levels	Normal	Normal
Diagnostic modality	Sonogram, magnetic resonance angiography	Computed tomography angiography and magnetic resonance angiography
Treatment plan	Surgery	Surgery
Postsurgical outcomes and follow up	Clinical resolution of symptoms and weight gain	Clinical resolution of symptoms and weight gain

Case one

A 36-year-old Caucasian female complained of weight loss of about 8 pounds in five months, recurrent vomiting, postprandial epigastric pain, and fatigue. The patient described the mild epigastric pain as crampy, lasting for a few minutes, and resolving independently, without associated diarrhea or constipation. Vomiting was non-bilious, not blood-stained, and non-projectile, containing recently ingested food. The patient had previously consulted a primary care physician who worked her up for possible gastritis, ruling out ulcer diseases. With a body mass index (BMI) of 28 kg/m^2^, the physical examination was unremarkable except for mild epigastric tenderness. Initial laboratory workup was within normal limits, including pregnancy test, amylase, and lipase. The *Helicobacter pylori* workup was negative. The stool guaiac was negative. The patient was managed empirically with proton pump inhibitors and diet modifications that favored high-fiber diets and was sent for an ultrasound that returned negative for intra-abdominal pathology. The patient’s symptoms persisted, particularly postprandial pain that necessitated a return to the clinic. At the subsequent visit, the patient’s weight loss continued with an additional 6 pounds from the previous visit. Physical examination and laboratory workup remained unremarkable except for a mild reduction in hemoglobin levels. The patient was also sent for an esophagogastroduodenoscopy, which showed mild mucosal erythema. Other extensive workup results were negative, including liver enzymes, cardiac enzymes, coagulation studies, erythrocyte sedimentation rate (ESR), celiac antibody panel, anti-gliadin, anti-endomysia antibodies, and stool analysis.

**Figure 1:**
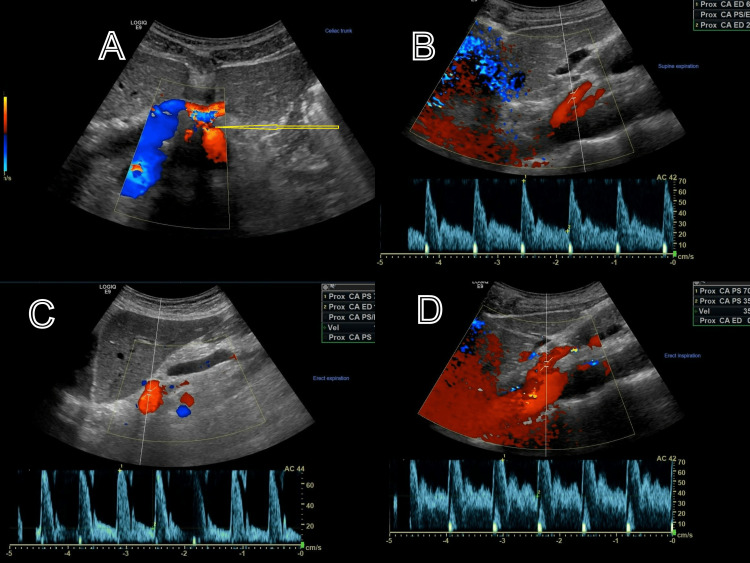
Doppler Ultrasound with the arrow showing the point at the constriction of the different variations of flow through the celiac trunk. A: constricted portion of the celiac artery. B: supine expiration. C: erect expiration. D: erect inspiration.

The patient’s primary symptoms of postprandial epigastric pain and early satiety persisted, necessitating a computed tomography (CT) scan of the abdomen and pelvis that returned normal. The functional sliding hernia was suspected, keeping vasculo-coagulative disorder in view. A barium swallow and manometry were performed, and a repeat ultrasound, this time with a Doppler, was completed. While barium swallow and manometry returned normal and the sonogram showed a normal liver and biliary tract, an evaluation of the vascular blood flow in the celiac artery detected a significant variation between the peak systolic velocity (PSV) during inspiration and expiration when the patient was erect and supine, respectively (Figure 1); 301 cm/second on inspiration, 276 cm on expiration while supine, and 142 cm/second and 128 cm/second on inspiration and expiration during erect positions, respectively (normal variants of celiac artery PSV of 67-187 with an SD of 28-161). PSV readings were near normal while the patient was sitting erect but substantially increased when he was supine.

**Figure 2:**
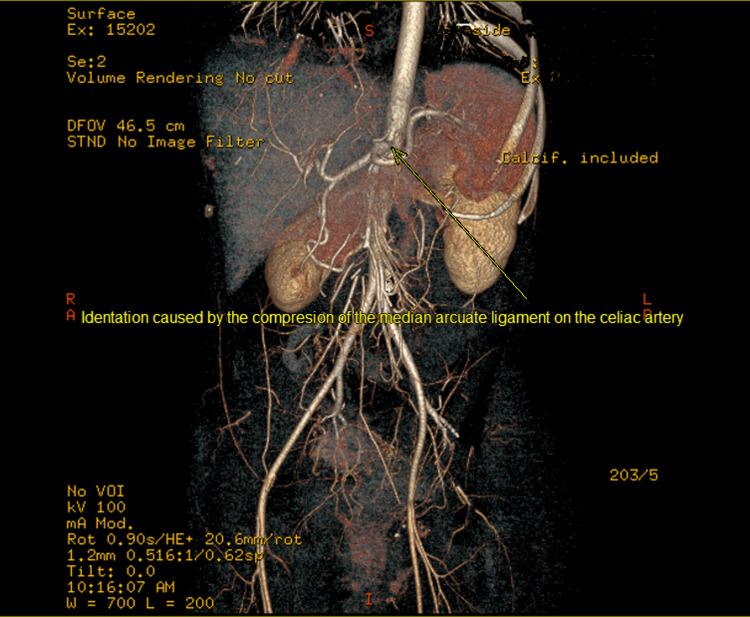
MRA showing the site of compression with pre- and post-ballooning of the celiac trunk. MRA: magnetic resonance angiography

As further assessment was required, a magnetic resonance angiography (MRA) of the mesenteric arteries was ordered. The MRA revealed characteristics of celiac artery compression near the hypothesized anatomical site of the diaphragm crux, probably due to MAL compression. There was both pre- and post-stenotic dilatation, showing the distinctive hook look of the MAL compression (Figure 2), while the remainder of the mesenteric vasculature was apparently normal. The patient was diagnosed with MALS and sent to a surgeon for further assessment and potentially surgical therapy. Following that, the patient had a laparoscopic release of her celiac artery, which involved dividing the MAL fibers that restrict the celiac artery. The post-surgery outpatient follow-up was uncomplicated, and the patient is now symptom-free and doing well.

Case two

**Figure 3:**
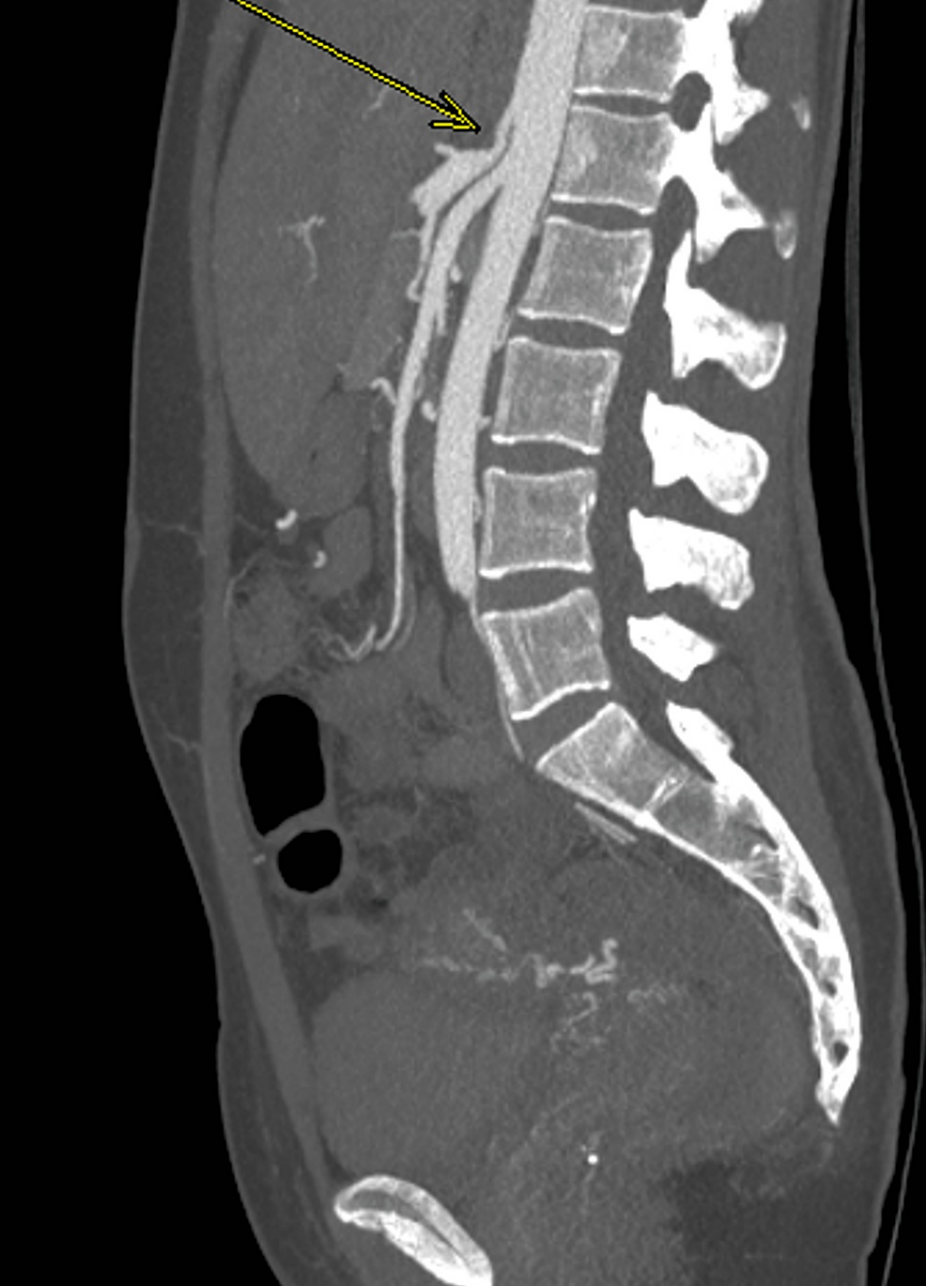
CTA showing compression of the celiac trunk as indicated by the arrow. CTA: computed tomography angiogram

A 29-year-old white female with a medical history of gastroesophageal reflux disease and well-controlled diabetes mellitus presented with a history of severe, constant, recurring epigastric pain. The index pain began three days prior to the presentation at the emergency department. The patient had a history of recurrent epigastric pain after meals with associated nausea and non-bilious vomiting, which progressively worsened, prompting a visit to the emergency room. The patient had seen several gastroenterologists about the problem. She had undergone repeated esophageal duodenoscopy, which did not show any significant abnormal findings. She admitted to a weight loss of roughly 30 pounds over three years due to decreased appetite. The patient was admitted to the emergency room for a physical examination and workup. With a BMI of 32 kg/m^2^, pertinent physical examination findings included dehydration, epigastric tenderness, and hyperactive bowel sounds. Other tests were unremarkable. Other laboratory blood workup results were not contributory besides anemia and mild hypokalemia. In addition, the patient underwent an ultrasound of the gallbladder that was negative for any concerns. A chest X-ray (anteroposterior) was negative. The Hepatobiliary iminodiacetic acid (HIDA) scan was unremarkable. The gastric emptying study was negative. Occult stool for blood was negative. Symptomatic management was started as follows: nausea with Reglan, rehydration, electrolyte replacement with intravenous (IV) fluids, pain medications, and proton pump inhibitors. Symptomatic management improved her symptoms, but they returned when tapered off. CTA/MRA of the abdomen and pelvis with IV contrast was normal for other abdominal organs but positive for compression of the celiac trunk (Figure 3). The patient was eventually referred to a surgeon for operative treatment of her celiac artery compression or MALS. The patient returned several months later to the outpatient department after postsurgical laparoscopic management, with a postsurgical diagnosis of MALS. The patient is currently symptom-free, has gained weight, and doing well.

## Discussion

As in the patients above, MALS is a disorder caused by the MAL compressing the celiac trunk. A quick review of the relationship between the MAL and the celiac trunk reveals that the MAL is the fibrous ligament that joins the diaphragmatic crura on both sides of the aortic foramina, creating the anterior edge of the aortic foramina. The celiac trunk is a major blood vessel that branches out from the aorta, with origins approximately lying below the MAL at around the level of the thoracic spine 11-12 and the first and second lumbar spines (T11-12 and L1/L2) [1], sending branches that supply most of the foregut structures of the gastrointestinal system such as the liver, spleen, and stomach. Historically, the anatomical theories of events of celiac artery compression were first documented in 1917 by Lipshutz [2], who noticed in cadaveric dissections that the celiac artery was sometimes overlapped by the diaphragmatic crura [2]. Then, in living humans, the syndrome was initially theorized in the 1960s, and it has been seen most often among thin women between the ages of 20 and 40 [1-10]. In 1963, Harjola [3] reported on the clinical resolution of postprandial epigastric pain and epigastric bruit in a 57-year-old man [3]. The pain associated with MALS has a neuropathic component [1-8,11] resulting from a combination of chronic compression and overstimulation of the celiac ganglion [11]. This compression of the celiac plexus may result in direct irritation of sympathetic pain fibers, causing splanchnic vasoconstriction that leads to ischemia [3-5,11]. Additionally, in patients with an obstructed or compressed celiac trunk, “vascular steal” of blood flow by bigger collateral arteries may diminish flow through the celiac trunk, resulting in symptoms of celiac artery compression [1,4,11]. MALS pain is also thought to be caused by either restricted blood flow via the celiac trunk, which may cause ischemia [1], or pain arising from the ganglionic compression of the nearby celiac plexus [3-5].

Epidemiology

As replicated in these two cases, it has been unexplainably observed that females exhibit significantly higher rates of this syndrome than males. A National Surgical Quality Improvement Program (NSQIP) study [6] that looked at MAL release in the United States between 2010 and 2020 found that out of 763 patients identified, the majority were white, non-Hispanic women with a mean age of 44 [6]. The frequency of occurrence in society is also variable, with some reports estimating about 7.3%. Moreover, this prevalence rate is compounded by incidental findings of patients who received abdominal imaging for other reasons [1,5-7]. Additionally, the risk factors have not been adequately defined [7]. In an attempt to characterize risk factors associated with this syndrome, Huynk et al. [8] reported the results as data from patients with MALS who underwent surgery between 2013 and 2018. In total, 11 patients were identified, of whom seven underwent diagnostics to evaluate gastric emptying. Five of these seven (71.4%) patients had abnormal anatomic visceral vasculature and, more likely, in young females aged 20-40.

Embryology and anatomy

On embryonic day 22, mesenchyme develops into the progenitor of the abdominal diaphragm [1], the septum transversum [8-10]. The septum transversum descends [1], reaching the level of the thoracic vertebrae by week eight [1,8-10] as a result of differential development between the anterior and posterior areas of the embryo. As muscular extensions that attach to the lateral surfaces of the lumbar vertebrae [1,8-10], the diaphragm’s two crura (legs) are a key structural component of the respiratory system [1]. The MAL is a tendinous band of fascia that connects the crura at their midpoint [1]. The abdominal aorta may pass through the diaphragm thanks to the aortic hiatus [1], a passageway between the diaphragm and the spinal column at T12 [1,8-10]. When the respective pair of developing segmental arteries converges at the midline [1] of the abdominal aorta, the celiac trunk, superior mesenteric artery (SMA), and inferior mesenteric artery (IMA) are all formed at the same time [8-12] as the diaphragm [1]. The SMA and IMA [1] develop into the main arteries that supply the middle and lower digestive tracts, respectively; the celiac trunk expands to provide arterial blood to the foregut [1,8-10]. As the gut tube expands, the three unpaired visceral branches travel caudally until they reach their final vertebral position by the end of the second month [1,8-11]. The most often reported adult vertebral sites for the celiac trunk, SMA, and IMA are T12, L1, and L3, respectively [8-12]. Thus, the MAL limits the size of the aortic gap, which is about the same level as the celiac trunk’s vertebrae [1]. The celiac trunk may be impinged upon if the MAL happens to overlap it [1]. The MAL is a fascial structure of the diaphragm that joins the crura on the right and left sides [1,8-11]. The celiac trunk is partly covered by the diaphragm [1,2] was noted as early as 1917, but the precise link between the celiac trunk and the MAL was not properly researched until quite recently.

The celiac trunk’s anterolateral sides are lined by collateral sympathetic ganglia, which are known as the celiac ganglia [1]. Providing sympathetic innervation to the foregut organs, they are the most prominent sympathetic ganglia in the body. Preganglionic sympathetic fibers from T5 to T12 reach the celiac plexus [1], which connects up to five celiac ganglia. Because the celiac ganglia are responsible for sympathetic innervation [1] and foregut visceral pain, ganglia impingement may lead to a wide range of symptoms, such as foregut pain that radiates, nausea, vomiting, epigastric fullness, and delayed gastric emptying [1,8-11].

Pathophysiology of symptoms

The two cases presented classically with the historically reported vague collection of symptoms associated with MALS, including epigastric pain that is postprandial in nature, vomiting, nausea, and weight loss [3,4-8,11]. Hence, a variety of manifestations due to tissue ischemia (resulting from reduced tissue blood flow) may make up for the MALS symptoms and/or neuropathic pain caused by celiac ganglion compression or overstimulation [1,3,4-9,11]. The vast majority of patients with partial CT compression are asymptomatic because collateral circulation typically prevents the development of symptoms. However, if the compression is severe and symptomatic, a diagnosis of MALS is considered [11]. These patients reported epigastric and right upper quadrant abdominal pain that worsened after meals, a common finding documented in various other published literature on MALS [3-8]. In addition to reduced calorie intake, associated with nausea and vomiting, fear of food-induced pain and delayed stomach emptying might worsen dietary intake and result in weight loss. Several additional illnesses, such as those causing gastroparesis, may produce postprandial vomiting and discomfort, but, fortunately, their stomach emptying investigations were normal. According to some research [12-14], about 75% of people in the emergency department had nausea or vomiting, about 50% were weak or lethargic, and about 35% had right upper abdomen pain [12-14]. In managing patients with a similar presentation, the need to explore other pathogenesis cannot be underscored in addition to MALS [12-14]. Another postulation for MALS is the aberrant stomach electrical rhythm (e.g., tachycardia) caused by celiac ganglion compression, referred to as the gastroparesis component [1,3-10]. These results point to the participation of celiac ganglion pathophysiology as a result of stomach neuromuscular function suppression [13-17].

Laboratory and radiological methods of diagnosis

In addition to incidental radiologic findings, targeted multiple imaging techniques, such as CTA, MRA, mesenteric arteriography, mesenteric duplex ultrasound, and gastric tonometry, may be utilized to show how the MAL is compressing the celiac trunk [11]. The choice of one modality over another depends on several factors, such as availability and cost-effectiveness. In working up these patients, clinicians bear in mind various other causes of upper gastrointestinal discomfort, such as vascular stenosis, ischemia, peptic ulcer disease, and pancreatitis, to name a few [7,8-15]. Imaging techniques such as CTA and MRA in breathing phases have demonstrated increased specificity and sensitivity [4-9] in visualizing external compression of the celiac axis with the characteristic attenuation of the outlines of the celiac trunk, as shown in this case series and many other documented instances [12-15]. However, because there have been accidental findings of celiac artery compression without symptoms [4-6], these imaging results may not be interpreted in isolation and are typically valuable when paired with the patient’s clinical presentation. Furthermore, in the positioning and breathing phases, mesenteric duplex ultrasonography may show an increase in peak systolic or end-diastolic pressures of blood flow through a markedly compressed celiac trunk as a consequence of constriction from external compression of the diaphragmatic crura or MAL [4-9,11-16].

Treatment modalities

Both patients had surgical interventions. Ligament release is performed [11] through open, laparoscopic, or robotic surgery. In certain schools of thought, celiac ganglionectomy and celiac artery revascularization are also encouraged [4-8,11-14]. Angioplasty may have limited usefulness because it does not treat the underlying extrinsic compression that causes symptoms; nonetheless, angioplasty with stenting may be utilized in resistant situations [11]. Doppler ultrasound and/or CT imaging are required for definitive diagnosis. Indication for surgery is established in these otherwise healthy, symptomatic patients by showing a baseline celiac velocity >200 cm/m^2^ and stenosis of the celiac artery on a CT scan or MRA [7,11,12].

**Figure 4:**
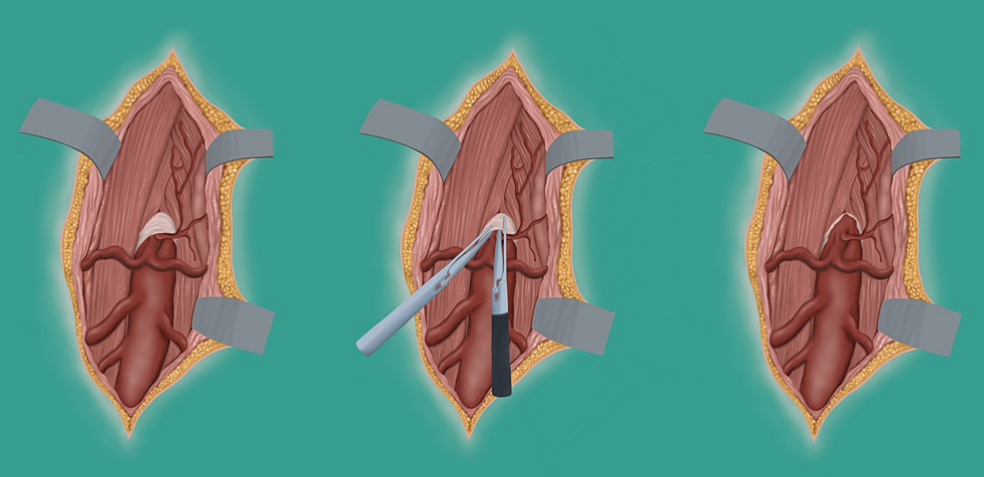
Artistic illustration or pre- and post-MAL release. This illustration is the creation of the authors’ imaginations, depicting anatomical connections between the celiac trunk and the MAL both pre and postoperatively. MAL: median arcuate ligament

Operative treatment with MAL release (see artistic illustration in Figure 4), first described in the 1960s by Harjola and Dunbar [6,15], has remained the standard treatment [4-7,9-14]. This surgery may be performed open, laparoscopically, or, more recently, robotically [1-6]. However, with the paucity of randomized clinical trials and data, there is insufficient evidence to help guide surgeons on the superiority of the different surgical approaches [4-7]. Some of these single-site retrospective investigations found that ligament release combined with celiac sympathectomy outperformed single-method ligament dissection, celiac sympathectomy, or arterial revascularization alone [4-7].

The aim of the surgery is to release the MAL, decompress the celiac artery, and release the celiac plexus [18]. Both traditional laparoscopy and robotic-assisted laparoscopy have been used with great success [18]. Intraoperative pre- and post-decompression flow velocity studies are performed routinely to assess the success of the procedure [18]. Postoperative angiography may be normal or can show some continuing minimal compression of the celiac artery (<30%) during expiration due to the chronicity of the condition inducing some arterial “stiffness” from intimal hyperplasia [18]. The first patient who underwent surgical release of the MAL demonstrated PSV on expiration reduced from its apparently elevated value pre-surgery, in keeping with other reported cases [18]. The celiac ganglion may be injected with xylocaine using an endoscopic ultrasound-guided method, and symptoms can be watched for improvement. Symptom relief boosts trust in the diagnosis, which influences the choice to have surgery [14-18]. Endoscopic ultrasound (EUS)-guided celiac block may be a predictor modality to measure response to surgical decompression [18].

Prognosis

Both of these patients made full recoveries. Data on patient prognosis are beginning to emerge as the quality of medical care continues to improve. A cure rate of about 80% has been postulated by some authors [4-6,16]. Factors that may influence prognosis have been postulated to include surgical skill and technique, availability of postsurgical follow-up imaging modalities, associated comorbidity, and other evolving parameters [1,2,4-6,17-18].

## Conclusions

The patients in this report had postprandial vomiting, stomach discomfort, nausea, and a negative workup for possible etiologies of upper gastrointestinal diseases other than MAL compression of the celiac trunk. Postoperative diagnosis of MALS was the subsequent outcome of the cases. These case reports demonstrate that a high suspicion index is required to diagnose MALS, especially in patients with recurrent postprandial pain, gastritis-like symptoms, and a negative EGD. Clinicians must be aware of this pathological entity, which is becoming more widely reported. Prompt diagnosis and treatment following suspicion of the syndrome are critical for resolving symptoms and improving patients’ quality of life. Imaging studies using high-resolution CT, MRI, or angiography are usually used to support the diagnosis, with particular attention paid to the morphology of the celiac trunk. Positional variations in Doppler flow rates of the celiac trunk may occasionally indicate external vascular pressures from the overlying ligament. So far, the recommended treatment modalities have primarily been surgical intervention, with little or no place for conservative management in symptomatic cases. Risk factors and prognosis are still evolving, and future research may aid in explaining these.
